# Vaginal biogenic amines: biomarkers of bacterial vaginosis or precursors to vaginal dysbiosis?

**DOI:** 10.3389/fphys.2015.00253

**Published:** 2015-09-29

**Authors:** Tiffanie M. Nelson, Joanna-Lynn C. Borgogna, Rebecca M. Brotman, Jacques Ravel, Seth T. Walk, Carl J. Yeoman

**Affiliations:** ^1^Department of Animal and Range Sciences, Montana State UniversityBozeman, MT, USA; ^2^Department of Microbiology and Immunology, Montana State UniversityBozeman, MT, USA; ^3^Institute for Genome Sciences, University of Maryland School of MedicineBaltimore, MD, USA; ^4^Department of Epidemiology and Public Health, University of Maryland School of MedicineBaltimore, MD, USA

**Keywords:** acid stress, polyamine, malodor, bacterial vaginosis, cadaverine

## Abstract

Bacterial vaginosis (BV) is the most common vaginal disorder among reproductive age women. One clinical indicator of BV is a “fishy” odor. This odor has been associated with increases in several biogenic amines (BAs) that may serve as important biomarkers. Within the vagina, BA production has been linked to various vaginal taxa, yet their genetic capability to synthesize BAs is unknown. Using a bioinformatics approach, we show that relatively few vaginal taxa are predicted to be capable of producing BAs. Many of these taxa (*Dialister, Prevotella, Parvimonas, Megasphaera, Peptostreptococcus*, and *Veillonella* spp.) are more abundant in the vaginal microbial community state type (CST) IV, which is depleted in lactobacilli. Several of the major *Lactobacillus* species (*L. crispatus, L. jensenii*, and *L. gasseri*) were identified as possessing gene sequences for proteins predicted to be capable of putrescine production. Finally, we show in a small cross sectional study of 37 women that the BAs putrescine, cadaverine and tyramine are significantly higher in CST IV over CSTs I and III. These data support the hypothesis that BA production is conducted by few vaginal taxa and may be important to the outgrowth of BV-associated (vaginal dysbiosis) vaginal bacteria.

## Introduction

The microbial community (microbiome) that colonizes the vagina of healthy women are typically dominated by one of several bacterial species of the genus *Lactobacillus* (Wolrath et al., 2002; Ravel et al., 2011; Macklaim et al., 2012). Therein, lactobacilli produce lactic acid creating an acidic environment (pH 2.8–4.2) that is inhospitable to many non-*Lactobacillus* commensals and potential vaginal pathogens (Amsel et al., 1983; Graver and Wade, 2011; O'Hanlon et al., 2011). This represents a classic form of niche-construction (Yeoman et al., 2011) recapitulated by human colonic and ruminal lactobacilli during gastrointestinal acidosis events (Allison et al., 1975; Bongaerts et al., 2000). Along with lactic acid, the lactobacilli may produce various antimicrobials (Aroutcheva et al., 2001; Anokhina et al., 2007; Rönnqvist et al., 2007) and toxin attenuating molecules (Cadieux et al., 2009; Li et al., 2011) that collectively are thought to constitute one of the primary barriers to vaginal diseases like bacterial vaginosis (BV) (Atassi and Servin, 2010).

BV is the most common disorder of the vagina in reproductive-aged women (Lefèvre et al., 1985) having been estimated to occur in almost one-third of U.S. women between 2001 and 2004 (Allsworth and Peipert, 2007). Clinical signs of BV include an amine or “fishy” vaginal odor, a creamy gray discharge, an elevated pH and/or the presence of superficial squamous cells with peripheral clumps of bacteria (clue cells) (Amsel et al., 1983). The signs are also observed alongside significant reductions in vaginal lactobacilli, which are replaced by an outgrowth of diverse, strict and facultative anaerobic bacterial taxa that commonly includes *Gardnerella vaginalis, Dialister* spp., *Atopobium* spp., *Prevotella* spp., *Mobiluncus* spp. and others (Spiegel et al., 1980; Amsel et al., 1983). These microbiological features may be causally linked as reductions in *Lactobacillus* spp. correspond to decreased vaginal concentrations of lactic acid and significant increases in vaginal pH (pH > 4.5) that provide a more hospitable environment for BV-associated species (O'Hanlon et al., 2011). However, while the depletion of vaginal lactobacilli and outgrowth of anaerobes is a characterizing co-feature of BV, it has been shown that ~27% of reproductive-age women exhibit vaginal microbiome deplete of *Lactobacillus* spp. (Ravel et al., 2011). This *Lactobacillus*-deplete vaginal microbiome is recognized as one of the five community state types (CSTs), termed by Ravel et al. as CST IV (Ravel et al., 2011) (Table 1). Recent findings have shown women with vaginal CST IV may persist in this state for extended periods of time without reporting symptoms of BV, regardless of their perception of whether those symptoms are present or not (Gajer et al., 2012). These findings may indicate a multi-staged, multi-microbial pathway to BV, whereby the protective features of the lactobacilli must be overcome prior to the chance exposure to, and colonization of less acid-sensitive organism(s) capable of eliciting additional clinical features (Lambert et al., 2013).

**Table 1:** **Community state types (CSTs) of the vagina**.

**I (*L. crispatus*)**		**II (*L. gasseri*)**		**III (*L. iners*)**		**IV (Diverse group)**		**V (*L. jensenii*)**	
**Women**	**pH**	**Women**	**pH**	**Women**	**pH**	**Women**	**pH**	**Women**	**pH**
27%	4.0 ± 0.3	6%	5.0 ± 0.7	34%	4.4 ± 0.6	27%	5.3 ± 0.6	5%	4.7 ± 0.4

*Adapted from Ravel et al. (2011)*.

**Figure 1:**
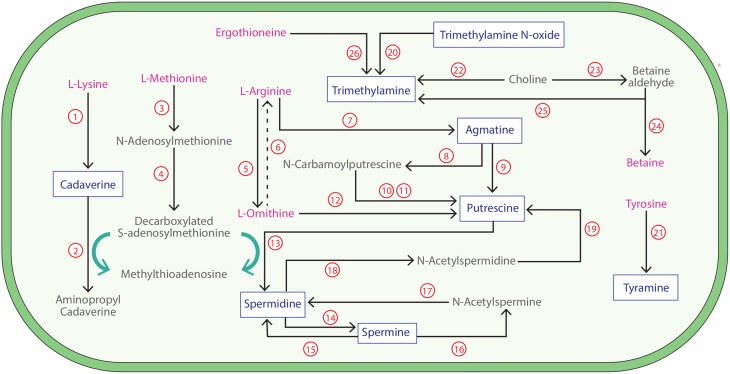
**Biogenic amine synthesis pathways in bacteria**. The dominant biogenic amine (BA) synthesis pathways involving the production of BAs (blue) from amino acids (pink) and associated derivatives in bacterial species are shown. Pathways are as follows (genes searched are shown in bold and underlined): **1. Lysine decarboxylase (Enzyme Commission number, E.C. 4.1.1.18)**; 2. Cadaverine aminopropyl-transferase (E.C. 2.5.1.-); 3. Methionine adenosyltransferase (E.C. 2.5.1.6); 4. S-adenosylmethionine decarboxylase (E.C. 4.1.1.50); **5. Arginase (E.C. 3.5.3.1)**; 6. Multiple enzyme reactions; **7. Arginine decarboxylase (E.C. 4.1.1.19)**; **8. Agmatine deiminase (E.C. 3.5.3.12)**; **9. Agmatinase (E.C. 3.5.3.11)**; **10. N-Carbamoylputrescine amidohydrolase (E.C. 3.5.1.53)**; 11. Putrescine transcarbamylase (E.C. 2.1.3.6); **12. Ornithine decarboxylase (E.C. 4.1.1.17)**; **13. Spermidine synthase (E.C. 2.5.1.16)**; **14. Spermine synthase (E.C. 2.5.1.22)**; **15. Spermine oxidase (E.C. 1.5.3.16)**; 16. Spermine acetyltransferase (E.C. 2.3.1.57); 17. Acetylpolyamine oxidase (EC 1.5.3.13); 18. Spermidine acetyltransferase (E.C 2.3.1.57); 19. Acetylpolyamine oxidase (E.C.1.5.3.13); **20. Trimethylamine N-oxide reductase (E.C. 1.7.2.3)**; **21. Tyrosine decarboxylase (E.C. 4.1.1.25)**; **22. Choline trimethylamine-lyase (E.C. 4.3.99.4)**; 23. Choline dehydrogenase (E.C. 1.1.99.1); 24. Betaine aldehyde dehydrogenase (E.C. 1.2.1.8); **25. Betaine reductase (E.C. 1.21.4.4)**; **26. Ergothionase (E.C. not applicable)**.

One clinical feature of BV, malodor, has been linked to increases in vaginal biogenic amines (BAs), including the polyamines putrescine, cadaverine, and trimethylamine (Yeoman et al., 2013). BAs are organic compounds with one or more amine (NH_2_) group(s), and may represent useful biomarkers of BV (Blankenstein et al., 2015). Other common BAs include tyramine, agmatine, spermine, and spermidine, the latter having also been observed at low relative concentrations in the vaginal metabolome (Yeoman et al., 2013). BAs are primarily produced via specific amino acid decarboxylation pathways (Shah and Swiatlo, 2008) (Figure 1). In *Escherichia coli* and many *Pseudomonas* species, putrescine is synthesized from arginine or ornithine using one of two major pathways: (i) decarboxylation of arginine to agmatine by arginine decarboxylase (encoded by gene s*peA*; Enzyme Commission number, E.C. 4.1.1.19) and then to putrescine either directly by agmatinase (s*peB* gene; E.C. 3.5.3.11) or via N-carbamoylputrescine as catalyzed by agmatine deiminase (E.C. 3.5.3.12) and then N-carbamoylputrescine amidohydrolase (*AguB* gene; E.C. 3.5.1.53), or (ii) decarboxylation of ornithine to putrescine via ornithine decarboxylase (*speC* gene; E.C. 4.1.1.17) (Tabor and Tabor, 1985) (Figure 1). These two putrescine biosynthesis pathways have been shown to operate simultaneously in many bacteria (Tabor and Tabor, 1985; Craciun and Balskus, 2012). Cadaverine and tyramine biosynthesis is less commonly described among bacterial species, although this could be a reflection of the relatively limited investigations in non-model species. *E. coli* synthesizes cadaverine during anaerobic growth at low pH in the presence of its precursor, lysine, as catalyzed by lysine decarboxylase (*cadA* gene; E.C. 4.1.1.18) (Watson et al., 1992). Tyramine is synthesized by various *Enterococcus* species by the decarboxylation of tyrosine (Fernandez de Palencia et al., 2011). Perhaps the best-studied BA in the context of BV is trimethylamine (TMA). TMA is most commonly produced by the reduction of trimethylamine oxide (TMAO), a reaction catalyzed by trimethylamine N-oxide reductase (E.C. 1.7.2.3). TMA can also be synthesized from choline by choline trimethylamine-lyase (Craciun and Balskus, 2012), N,N,N-trimethylglycine via betaine reductase (E.C. 1.21.4.4), and ergothioneine by ergothionase (Muramatsu et al., 2013). A previous study showed strains of vaginal *Mobiluncus* species, including both *M. mulieris* and *M. curtisii* were able to produce TMA through the reduction of TMAO, and weakly through the reduction of choline (Cruden and Galask, 1988). While various bacterial species have been shown to be capable of producing BAs, aside from *Mobiluncus* spp., and the vaginal parasite, *Trichomonas vaginalis*, which has been shown to encode an ornithine decarboxylase (Yarlett et al., 1993), little is known about their production by the vaginal microbiome.

**Figure 2:**
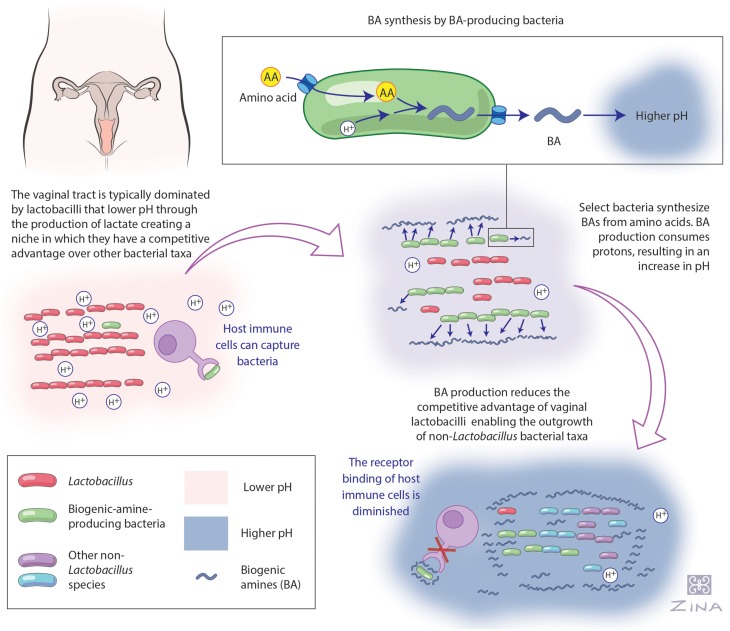
**Hypothetical model of biogenic amine production and its effect on the vaginal ecosystem**. This figure summarizes our hypothetical model of the role of biogenic amine (BA) production and it's influence on the vaginal ecosystem as outlined in this article. Beginning at the top left corner, women with a *Lactobacillus*-dominated vaginal microbiome maintain a low pH via their production of lactic acid, which excludes non-lactobacilli (O'Hanlon et al., 2011, 2013; Ravel et al., 2011). Following the arrow to the right, it is well established in the literature that model species, such as *E. coli*, use amino acid decarboxylation as an acid stress resistance strategy (Recsei and Snell, 1972; Large et al., 2005; Kanjee and Houry, 2013). BA production consumes protons, increasing the pH of the local habitat (Recsei and Snell, 1972; Meng and Bennett, 1992; Soksawatmaekhin et al., 2004), which we suggest occurs in the vagina. Following the arrow to the bottom right, we suggest that together these changes promote colonization by BV-associated bacteria and may additionally reduce the host's immune defenses as has previously been identified in the human urogenital pathogen *Neisseria gonorrhoeae* and the BA, spermine (Goytia and Shafer, 2010). Illustrated by Zina Deretsky.

In addition to their potential as biomarkers of BV, BAs may also be important to facilitating the outgrowth of BV-associated vaginal taxa. This hypothesis is based on the following observations: (i) amino-acid decarboxylation involves the consumption of intracellular hydrogen ions and is a well-described bacterial acid resistance and mitigation mechanism (Kanjee and Houry, 2013); and (ii) the growth and resistance to host immunological defenses of some bacteria, including the urogenital pathogen, *Neisseria gonorrhoeae*, has been shown to be improved in the presence of various BAs (Strøm et al., 1979; Goytia and Shafer, 2010; Nasrallah et al., 2011; Jelsbak et al., 2012). It is also noteworthy that BAs have been correlated with numerous host disease states (Löser et al., 1990; Paik et al., 2006; Pegg, 2009; Brooks, 2013). Here we put forward a novel conceptual hypothesis of vaginal dysbiosis via bacterial BA-production (detailed in Figure 2) that precedes BV development.

## Methods

### Strains and genomes

Our search database included 50 urogenital isolates whose genomic data was available in GenBank (http://www.ncbi.nlm.nih.gov/genbank/). Taxonomy was guided toward the major taxa identified by Ravel et al. (2011) in their comprehensive evaluation of the vaginal microbiome of reproductive age women, and taxa in the studies of Yeoman et al. (2013) and Srinivasan et al. (2012) as being prominent and associated with odor or BA production. Where representative genomes of vaginal isolates were not available, sequenced isolates sourced from other body niches were utilized as available (*n* = 7), or, if not available, all protein-coding sequences within that genus (*n* = 7) were utilized. For example, *Bifidobacterium dentium* Bd1 was sequenced as part of the Human Microbiome Project, but previous literature has shown four other *Bifidobacterium* species are commonly isolated from the vagina (Korshunov et al., 1999). Therefore, protein-coding data of gastrointestinal isolates of *Bifidobacterium bifidum, B. breve, B. adolescentis*, and *B. longum* were used. Elsewhere, no complete genomic data or inventory of vaginal *Sneathia* species/isolates have been reported, so we utilized all available *Sneathia* protein coding data in GenBank. All available protein-coding sequences of selected bacterial taxa were downloaded from GenBank.

### Identification of biogenic amine producing genes in vaginal bacteria

Functionally characterized gene sequences of biogenic amine-synthesizing proteins (BSPs), including arginine decarboxylase, agmatine deiminase, N-carbamoylputrescine amidohydrolase, agmatinase, lysine decarboxylase, ornithine decarboxylase, tyrosine decarboxylase, trimethylamine N-oxide reductase, ergothionase, choline trimethylamine-lyase, betaine reductase, spermidine synthase, and spermine synthase (Table S1) were obtained from GenBank and used in a stand-alone BLASTP search against a database of the protein coding sequences of vaginal taxa. The BA-synthesizing protein sequences used in the study are available in the Supplementary Materials. BLASTP alignments with ≥45% sequence identity across ≥80% of the query sequence, or ≥35% sequence identity across ≥90% of the query sequence with an expected value (*E*-value) ≤ 10^−30^ were determined to be a positive indication of BSPs among the genetic infrastructure of the query microbe. All protein sequences were also searched using hidden markov models (HMMs) corresponding to the BSPs downloaded from the TIGRFAM (http://www.jcvi.org/cgi-bin/tigrfams/index.cgi) or Pfam (http://pfam.xfam.org/) databases (Table S1) using HMMer (http://hmmer.janelia.org/). HMM hits that had *E*-values below their prescribed trusted cut-offs were determined to be a positive indication of these genes in the query protein sequences. Data were visualized using heat maps that were produced using the *gplots* package (Warnes et al., 2009) constructed in R v.3.1.2 (Ihaka and Gentleman, 1996). Results were color coded in the heat map based on the extent of evidence supporting the presence of each BSP in each respective genome.

### Determining biogenic amine producing taxa among CSTs with stratification by pH

Data on the fine scale taxonomic composition and vaginal pH of 394 women were obtained from Ravel et al. (2011). Taxa corresponding to those identified as encoding putative BSPs were interrogated. The Ravel dataset was stratified by CST and pH so that the relative abundances of the 16S rRNA gene sequence reads from BSP-encoding taxa were averaged for each of the five CSTs and then further categorized into ether low or high pH. We determined pH 4.0–4.5 was “low” and pH 4.6–7.0 was “high,” based on pH ranges used to diagnose BV based on Amsel's criteria (Amsel et al., 1983). Significance between groups was determined with the Mann-Whitney-Wilcoxon test with Bonferroni correction used for multiple testing where *p* < 0.05 performed in R v.3.1.2 (Ihaka and Gentleman, 1996).

### Threshold analysis of vaginal taxa along the pH gradient

To further understand the association of BSP-encoding taxa with pH in the vagina, threshold indicator taxa analysis (TITAN) was conducted. TITAN was used to assess the association of taxa to pH using the dataset obtained from Ravel et al. (2011). TITAN uses IndVal (Indicator Value) scores to capture the strength-of-association between a particular species along a gradient (Dufrene and Legendre, 1997). The probability of obtaining an equal or larger IndVal score from random data is estimated by comparing the magnitude of each observed IndVal score with those generated by randomly assigning membership along the variable gradient via permutations. Bootstraps were used to compute the confidence interval of the change point location along the gradient for each taxa. Confidence intervals for each IndVal were generated using 500 permutations of the data. The bacterial abundance dataset was log10(x + 1) transformed prior to analysis to reduce the influence of highly variable taxa and rare operational taxonomic units (OTUs) (< 5 occurrences across women) were removed (Baker and King, 2010). TITAN analyses were performed using the *mvpart* package in R v.3.1.2 (Ihaka and Gentleman, 1996) with code provided in Baker and King (2010).

## Determining biogenic amine levels corresponding to CSTs

### Sample set for measuring biogenic amine levels

Analyses were performed on self-collected mid-vaginal swab (Copan flocked nylon elution-swab and Starplex double headed rayon swab) samples collected from 37 non-pregnant, non-lactating women, aged 18–45 years recruited for a single visit to the Center for Health Behavior Research (CHBR) at the University of Maryland School of Public Health (UMSPH) as part of a previously published study (Brotman et al., 2014). Women had to be healthy as determined by medical history, with absence of acute or chronic illnesses. In addition, participants were excluded if they had used an antibiotic or anti-mycotic in the prior 30 days or reported a known history of other drug or alcohol dependence in the prior 12 months. All participants provided written informed consent. Ethical approval was obtained from the Institutional Review Boards of the University of Maryland Baltimore (UMB) and the UMSPH.

### Sample preparation

Samples were eluted from swabs in sterile molecular water and subjected to both gas chromatography mass spectrometry (GC/MS) and liquid chromatography mass spectrometry (LC/MS) with Orbitrap Elite accurate mass platforms (Thermo Scientific, Waltham, MA, USA). Sample processing was performed by Metabolon (Durham, NC, 27713) using an automated MicroLab STAR® system (Hamilton Company, Reno, NV, USA). Recovery standards were added prior to the first step in the extraction process for QC purposes. Sample preparation was conducted using a proprietary series of organic and aqueous extractions to remove the protein fraction while allowing maximum recovery of small molecules. The resulting extract was divided into two fractions: one for analysis by LC and one for analysis by GC. Samples were placed briefly on a TurboVap®(Zymark, Hopkinton, MA, USA) to remove the organic solvent. Each sample was then frozen and dried under vacuum. Samples were then prepared for the appropriate instrument, either LC/MS or GC/MS.

### Liquid chromatography mass spectrometry

LC/MS measurements were conducted on a Waters ACQUITY ultra-performance liquid chromatography (UPLC) and a ThermoFisher Scientific Orbitrap Elite high resolution/accurate mass spectrometer, which consisted of a heated electrospray ionization (HESI) source and Orbitrap mass analyzer operated at 30,000 mass resolution. The sample extract was dried then reconstituted in LC-compatible solvents, each of which contained eight or more injection standards at fixed concentrations to ensure injection and chromatographic consistency. One aliquot was analyzed using acidic positive ion optimized conditions and the other using basic negative ion optimized conditions in two independent injections using separate dedicated columns. Extracts reconstituted in acidic conditions were gradient eluted using water and methanol containing 0.1% formic acid, while the basic extracts, which also used water/methanol, contained 6.5 mM ammonium bicarbonate. The MS analysis alternated between MS and data-dependent MS^2^ scans using dynamic exclusion. Raw data files were archived and extracted as described below.

### Gas chromatography mass spectrometry

Samples for GC/MS analysis were re-dried under vacuum desiccation for a minimum of 24 h prior to being derivatized under dried nitrogen using bistrimethyl-silyl-triflouroacetamide (BSTFA). The GC column was 5% phenyl and the temperature ramp is from 40 to 300°C in a 16 min period. Samples were analyzed on a Thermo-Finnigan Trace DSQ fast-scanning single-quadrupole mass spectrometer using electron impact ionization. The instrument was tuned and calibrated for mass resolution and mass accuracy prior to use. The information output from the raw data files was automatically extracted as described below.

### Data extraction

The data extraction of the raw MS data files yielded information that could be loaded into a relational database and manipulated without resorting to binary large object (BLOB) manipulation. Once in the database the information was examined and peaks were identified using Metabolon's proprietary peak integration software, and component parts were stored in a separate and specifically designed complex data structure (Ryals et al., 2007).

### Compound identification

Spectra corresponding to each BA were identified by comparison to library entries of purified BA standards and their distinction from more than 1000 other commercially available purified standard compounds. The combination of chromatographic properties and mass spectra gave an indication of a match to the specific BA compound or an isobaric entity. Results were manually curated to ensure that data were accurate and to remove any system artifacts, mis-assignments, and background noise.

## Results

### Distribution of biogenic amine synthesizing proteins among common vaginal taxa

**Figure 3:**
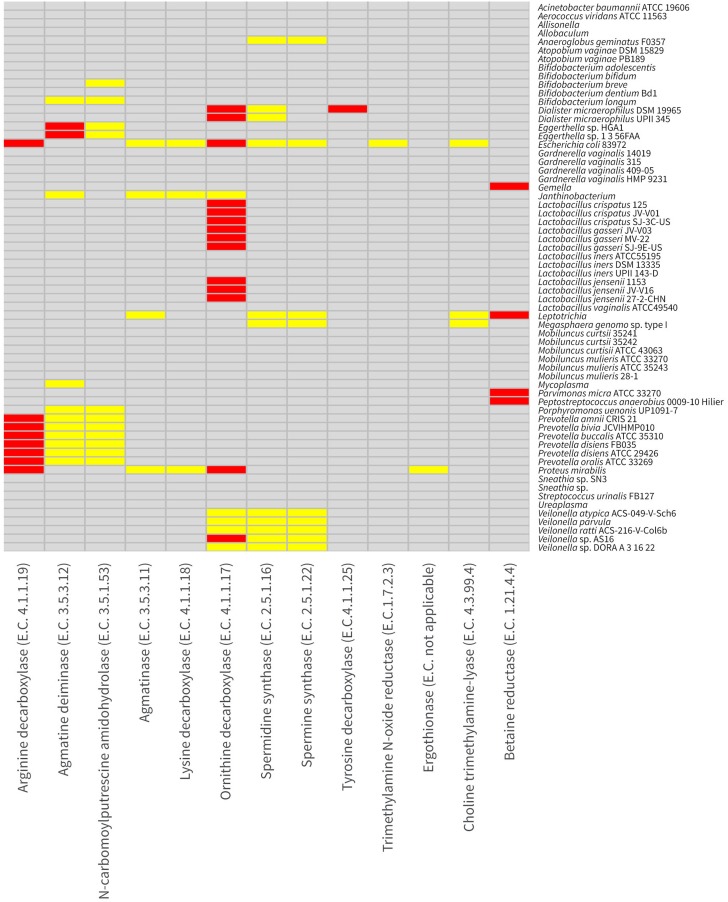
**Distribution of biogenic amine-synthesizing proteins (BSPs) in vaginal taxa**. The distribution of BSPs in the 65 vaginal taxa examined is presented as a heat map. Gray indicates no evidence of the BSP shown on the horizontal axis in the taxon shown on the vertical axis. Yellow indicates a strong alignment to a characterized homolog or to a corresponding hidden markov model (HMM), while red indicates both a strong alignment to a characterized homolog and a corresponding HMM. Enzyme numbers (E.C.) are shown with BSPs.

Previous studies have shown correlative relationships among the vaginal odor characteristic of BV, BAs including putrescine, cadaverine, and trimethylamine (TMA) and particular bacterial taxa (Chen et al., 1979, 1982; Sanderson et al., 1983; Cruden and Galask, 1988; Wolrath et al., 2002; Srinivasan et al., 2012; Yeoman et al., 2013). To examine the potential for common vaginal taxa to produce these BAs, we examined the protein coding genes of 65 common vaginal bacterial taxa for the presence of biogenic BSPs by alignment to functionally characterized BSPs and searches with hidden markov models (HMMs) built for BSPs (see Methods). Interestingly, very few vaginal taxa possessed BSPs for the biosynthesis of the major BAs (Figure 2). *E. coli* 83972 was the only taxa predicted to be capable of producing putrescine, cadaverine, and TMA (Figure 3). *E. coli* 83972 also appeared to be capable of producing agmatine, spermine and spermidine. All examined strains of *Dialister micraerophilus, Veilonella* spp., *Proteus mirabilis, Janthinobacterium* spp. and common lactobacilli, *L. crispatus, L. gasseri*, and *L. jensenii* (but not *L. iners*) each encoded BSP homologs of genes capable of producing putrescine through the decarboxylation of ornithine (Figure 3). *Prevotella amnii, P. bivia, P. buccalis, P. disiens, P. oralis* possessed putative BSPs allowing the production of agmatine, and eventually putrescine through the decarboxylation of arginine. Two *Eggerthella* spp. *B. longum, B. breve, Mycoplasma* spp., *Porphyromonas uenonis, Parvimonas micra* and *Gemella* spp. also possessed one or more gene homologs of BSPs on the pathway conversion of arginine to agmatine and then putrescine. Only the taxa *E. coli* 83972, *P. mirabilis*, and *Janthinobacterium* spp. were found to possess putative BSPs encoding lysine decarboxylases for the biosynthesis of cadaverine (Figure 3). It was noteworthy that in each instance the lysine decarboxylase genes were incorrectly annotated as arginine decarboxylases. *E. coli* 83972, *Megasphaera genomosp type I, Anaeroglobus geminatus* F0357, along with all examined *Dialister microaerophilus, Veillonella* and *Leptotrichia* spp. possessed spermidine and/or spermine synthase genes. Spermidine and spermine synthase genes were not readily distinguishable from one another bioinformatically. *D. micraerophilus* DSM 19965 was the only taxon predicted to possess a tyrosine decarboxylase enabling the production of the monoamine tyramine.

The BA trimethylamine (TMA) is synthesized through four known pathways (Figure 1). Interestingly, only *E. coli* 83972 was found to possess a homolog of the trimethylamine N-oxide reductase, the only currently described enzyme capable of synthesizing TMA from trimethylamine N-oxide (TMAO) (Strøm et al., 1979). This was unexpected given previous findings that TMA was produced in cultures containing TMAO by vaginal *Mobiluncus* spp. (Cruden and Galask, 1988). To reconcile this discrepancy, we more intensively examined the genomes of the vaginal *Mobiluncus* isolates. Weaker alignments were found between the characterized TMAO reductase and a gene annotated in each *M. curtisii* genome as a dimethyl sulfoxide reductase α-subunit (28% protein identity, over 95% of the query, *E* = 1 × 10^−66^). Similar matches were not found in representative *M. mulieris* genomes and were not supported by HMM examinations. Broader searches among all available sequences from the *Mobiluncus* genus also did not reveal any candidate homologs. Additionally, available *Mobiluncus* spp. genomes did not encode a homolog of the choline trimethylamine-lyase required for the production of TMA from choline, also reported by Cruden and Galask (1988). However, homologs of BSPs encoding choline trimethylamine-lyase were observed in *E. coli* 83972, *Megasphaera genomosp type I*, and various *Leptotrichia* spp. TMA could also be synthesized by *P. micra, Peptostreptococcus anaerobius*, and various *Leptotrichia* and *Gemella* spp. using betaine reductase. *P. mirabilis* was the only taxon found to possess putative BSPs encoding ergothionase, which catalyzes conversion of ergothioneine to thiolurocanic acid and TMA (Craciun and Balskus, 2012).

### BSP-encoding taxa associated with increased pH and CST IV

**Figure 4:**
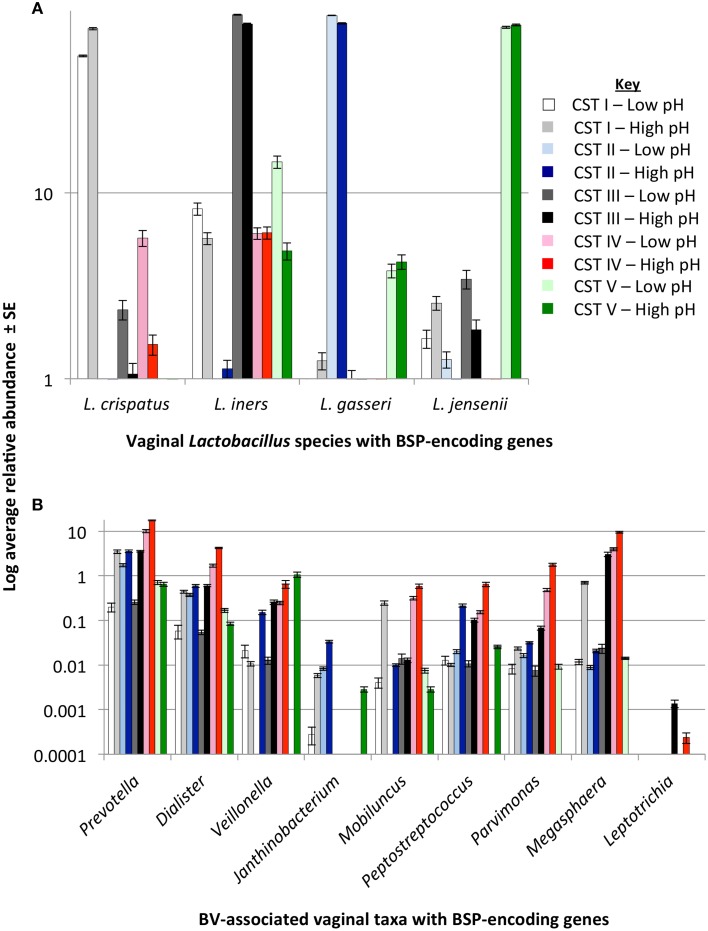
**BSP-encoding gene presence stratified by community state type (CST) and pH**. Bar charts display average log scaled relative abundances ± standard error (SE) of bacterial taxa in the vaginal microbiome of healthy women with biogenic amine-synthesizing proteins (BSP) encoding genes as identified from GenBank. Taxa are grouped by *Lactobacillus* species **(A)** and BV-associated anaerobes **(B)** shown in relation to CST and vaginal pH. Abundance data was taken from Ravel et al. (2011). Significant differences between groups are shown in **Table 2**.

To determine the relationship of the various BSP-possessing taxa identified above to the growth of BV-associated bacteria, we tested their relative abundances in the various CSTs using data provided by Ravel and colleagues on 394 reproductive-aged women (Ravel et al., 2011). Because *Mobiluncus* spp. had previously been shown to produce TMA (Cruden and Galask, 1988), we also included this taxa in our analyses. BSP-encoding taxa were present in vaginal microbiome from all CSTs. However, there was a clear difference in the abundance and type of BSP-encoding taxa between CSTs (Figure 4). Women categorized into the low-*Lactobacillus* CST IV, had higher levels of predicted putrescine-producers, *Prevotella, Dialister*, and *Veillonella* spp. and TMA producers, *Peptostreptococcus, Parvimonas*, and *Megasphaera* spp. as well as *Mobiluncus* spp. (Figure 4).

As pH is considered an important barrier to the outgrowth of BV-associated bacteria, and the production of BAs may impact pH directly, we then stratified the data for each CST by pH (pH 4.0–4.5 “low” and pH 4.6–7.0 “high”) and re-examined the relative abundances of each BSP-encoding taxa. These pH categories were determined based on the known pH ranges of CST groups representing women (Table 1) (Ravel et al., 2011) and from current clinical criteria (Amsel et al., 1983). *Prevotella* and *Dialister* spp. were significantly more abundant in CST I, III, and IV when pH was 4.6 or greater (Figure 4, Table 2). In the “high” pH category for CST III, *Veillonella, Parvimonas* and *Megasphaera* species were also significantly more abundant (Figure 4, Table 2). As expected, *Lactobacillus crispatus, L. gasseri, L. iners*, and *L. jensenii* displayed a decreasing trend with increasing pH while BV-associated BSP-encoding taxa displayed the opposite trend (Figure S1).

**Table 2:** **Difference in low and high pH for BSP-encoding taxa by CST**.

**Species/Genus**	**Testing between low and high pH groups**				
	**CST I**	**CST II**	**CST III**	**CST IV**	**CST V**
*L. crispatus*					
*L. iners*					
*L. gasseri*					
*L. jensenii*					
*Prevotella*			^**^		
*Dialister*	^*^		^**^		
*Veillonella*					
*Janthinobacterium*			NA	NA	NA
*Mobiluncus*					
*Peptostreptococcus*					
*Parvimonas*			^*^		
*Megasphaera*			^**^		
*Leptotrichia*	NA	NA		NA	NA

*The presence of biogenic amine-synthesizing proteins (BSPs) obtained bioinformatically as grouped by community state types (CSTs). Results obtained from Mann-Whitney-Wilcoxon testing with Bonferroni corrections between groups as shown in Figure 2*.

** P < 0.01;

* *P < 0.05*.

*P-values were corrected for multiple testing. Not applicable due to insufficient observations (NA)*.

**Figure 5:**
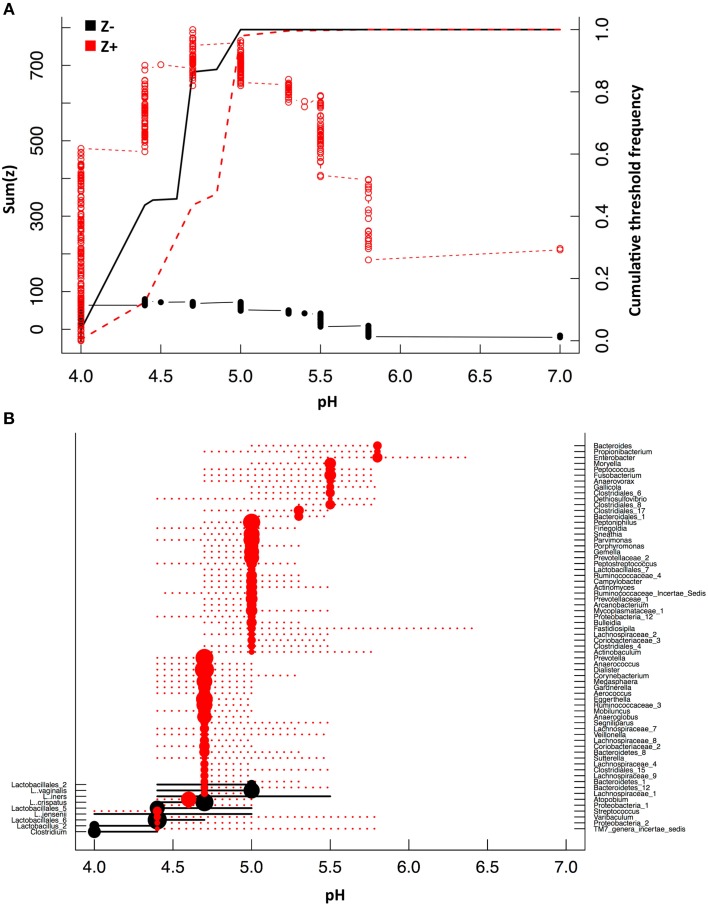
**Threshold indicator taxa analysis (TITAN) with pH**. Community thresholds as determined by TITAN. The cumulative sum of Z− (taxa responding negatively to higher levels of pH) and Z+ (taxa responding positively to higher levels of pH) scores are displayed in relation to pH **(A)**. Change points (dots) and 95% and 5% confidence intervals (dotted lines) for taxa along the pH gradient are displayed **(B)** (*P* < 0.05, purity > 0.07, reliability > 0.7 for 500 bootstrap and 250 permutation replicates). Size of change point symbol (dots) is proportional to the magnitude of the taxa response.

The relationship between pH and vaginal taxa was further explored with TITAN. TITAN provides an understanding of the community response to gradients in the surrounding habitat and can be used to assess community thresholds. We compared vaginal taxa along the gradient of pH observed in the vagina of 394 women and identified the value of greatest synchronous decline in bacterial taxa occurs at pH 4.4 (Figure 5, Table 3). The individual taxa that contributed strongly and negatively to increases in pH included all of the dominant *Lactobacillus* spp. and with the exception of a *Clostridium* spp. were the only genera that had significantly negative decline with increases in pH (Figure 5, Table S2). Those taxa which responded significantly and positively to higher pH included the BSP-encoding species from the genera *Prevotella, Dialister, Parvimonas, Megasphaera, Mobiluncus, Peptostreptococcus*, and *Veillonella* spp. (Figure 5, Table S2). In addition, species from the *Peptoniphilus, Anaerococcus, Atopobium, Sneathia*, and *Finegoldia* genera were more common where pH > 4.6 (Figure 5, Table S2).

**Table 3:** **Community level threshold indicator analysis (TITAN) for pH**.

	**pH thresholds**	**5%**	**50%**	**95%**
Sum z−	4.4	4.4	4.7	5.0
Sum z+	4.7	4.4	5.0	5.0

*Threshold indicator analysis (TITAN) identifies the response of vaginal bacteria in relation to pH to estimate a community threshold. The cumulative sums of z− (taxa responding negatively to higher levels of pH) and z+ (taxa responding positively to higher levels of pH) scores are displayed. The thresholds are based on the sum of the z− and z+. Associated percentiles correspond to the frequency distribution of thresholds from 500 bootstrap replicates. Abundance data was taken from Ravel et al. (2011)*.

### Putrescine and cadaverine are higher in CST IV vaginal microbiome

**Figure 6:**
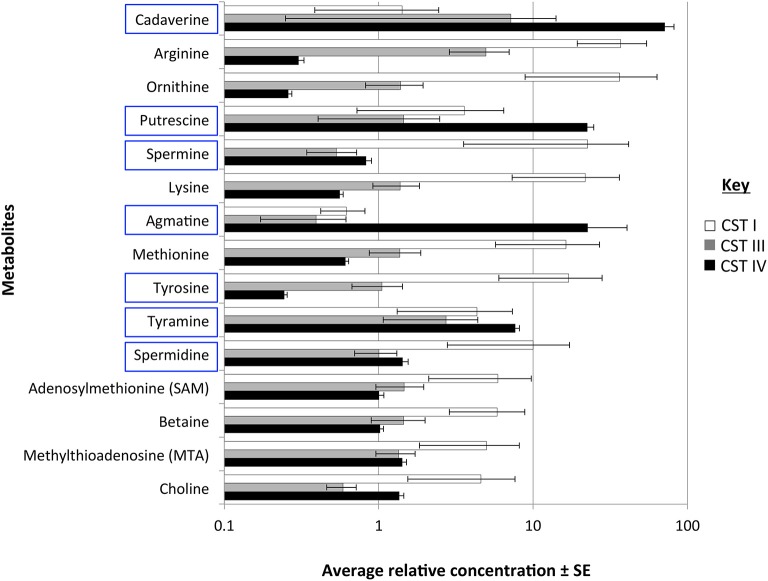
**Metabolite presence in the vagina associated with community state types (CSTS)**. Bar chart displays the log average relative concentration of metabolites ± standard error (SE) involved in biogenic amine biosynthetic pathways (as shown in Figure 1). BAs are highlighted in blue rectangles. Metabolites were measured from the vagina of 37 women. Bar colors indicate the CSTs of the vaginal microbiome. Significant differences between groups are shown in Table 4.

To determine the actual changes in vaginal BA levels associated with CSTs, we performed a cross-sectional study on the vaginal metabolome of 37 women found to represent the three most common CSTs (CST I, CST III, and CST IV). Women with CST IV had higher abundances of the BAs cadaverine, putrescine, and agmatine over the other CSTs (Figure 6, Table 4). All precursor amino acids and derivatives including the amino acids lysine, methionine, ornithine, arginine and tyrosine were lower in CST IV over CSTs I and III (Figure 6, Table 4). These trends in BAs and amino acids were significant between CST IV and CST I but not CST IV and CST III (Table 4). Additionally, the triamine, spermidine, and tetraamine, spermine, were higher in women with the CST I vaginal microbiome (Figure 6, Table 4). Our analyses did not detect TMA, although it was not clear if this was methodological rather than a true absence.

**Table 4:** **Difference in metabolite concentration between CSTs**.

**Metabolites**	**Testing groups**		
	**CST I, III**	**CST I, IV**	**CST III, IV**
Adenosylmethionine (SAM)			
Agmatine		^*^	
Arginine		^**^	
Betaine			
Cadaverine		^**^	
Choline			
Lysine		^**^	
Methionine		^**^	
Methylthioadenosine (MTA)			
Ornithine		^**^	
Putrescine		^**^	
Spermidine			
Spermine			
Tyramine			
Tyrosine		^**^	

*Difference observed between community state types (CSTs). Results obtained from Mann-Whitney-Wilcoxon testing with Bonferroni correction between groups as shown in Figure 6*.

** P < 0.01;

* *P < 0.05*.

## Discussion

BAs are produced by all eukaryotes but only by select bacteria (Pegg, 2013). They are compounds that have one or more amino group (−NH_2_) and are often derived from amino acid precursors (Tabor and Tabor, 1985). In bacteria, BAs are involved in many essential reactions relevant to transcription, translation, growth and metabolism (Tabor and Tabor, 1985; Wallace et al., 2003; Wortham et al., 2007). Recently it has been realized that BAs are associated with a number of other functions in prokaryotes specific to the manifestation and symptoms of infections, including improved acid resistance, protection from oxidative stress and host immunological defenses, promotion of biofilm formation, and control of membrane permeability (Tabor and Tabor, 1985; Dela Vega and Delcour, 1996; Jung and Kim, 2003; Patel et al., 2006; Wortham et al., 2007; Shah and Swiatlo, 2008; Goytia and Shafer, 2010; Zhao and Houry, 2010; Nasrallah et al., 2011; Jelsbak et al., 2012). Correspondingly, BAs have been associated with various host disease states (Löser et al., 1990; Paik et al., 2006; Pegg, 2009; Brooks, 2013). There is currently no consensus as to the common function of BAs in bacteria, yet their presence and production has been shown to be a factor in the manifestation of several bacterially mediated diseases. We hypothesize this may also be the case with BV (outlined in Figure 2).

BA synthesis as indicated by the presence of genes encoding BSPs were observed sporadically across vaginal taxa. BA synthesis is coupled to amino acid decarboxylation, which has been shown to be a protective mechanism developed to maintain the intracellular pH homeostasis of various bacteria (including *E. coli* and some *Lactobacillus* spp.) when growing under acidic conditions (Jung and Kim, 2003; Azcarate-Peril et al., 2004; Large et al., 2005; Cid et al., 2008; Kanjee and Houry, 2013). The decarboxylase enzymes are typically induced by the presence of the amino acid precursor when confronted with an acidic environment (Yarlett et al., 1993). The decarboxylation reaction consumes a proton, which cumulatively results in an increased pH within the cytoplasm creating a transmembrane pH gradient (Molenaar et al., 1993). The decarboxylase enzymes function in co-operation with an amino acid/BA antiporter system that moves the BAs to the outside of the cell in exchange for extracellular amino acids (Yarlett et al., 1993). This massive extrusion of the basic BA product coupled by the uptake of the amino acid substrate and proton motive force may also contribute to increases in the pH of the extracellular milieu (Meng and Bennett, 1992; Molenaar et al., 1993; Bearson et al., 1998). Two of these systems, well understood in *E. coli*, produce cadaverine and putrescine through the decarboxylation of lysine and ornithine, respectively (Kanjee and Houry, 2013). A number of studies have identified increased concentrations of these BAs and others in women with BV (Wolrath et al., 2002; Baker and King, 2010; Sakamoto et al., 2012; Lambert et al., 2013). Together, eight genera and multiple species including the most common *Lactobacillus* spp. encoded either lysine or ornithine decarboxylase or both. The BSP encoding ornithine decarboxylase was the most common system we identified across the 64 vaginal taxa investigated in our analysis and several other systems were common to multiple species within a genus. All vaginal lactobacilli with the exception of *L. vaginalis* and *L. iners* were identified as having ≥35% sequence identity to the ornithine decarboxylase protein sequences from non-vaginal *L. brevis* and *L. saerimneri* (formerly *Lactobacillus* sp. strain 30a) (Guirard and Snell, 1980; Romano et al., 2013). Like these two species, many foodborne lactobacilli have been identified as being capable of ornithine decarboxylase (Arena and Manca de Nadra, 2001; Pereira et al., 2001; Azcarate-Peril et al., 2004). These enzymes are likely to be used periodically as a way of overcoming acid stress. However, we suggest that non-lactobacilli are capable of stronger production, which may cause a feedback inhibition of the enzymes in the lactobacilli that reduces their ability to outcompete other species in acidic conditions. The literature suggests that these systems operate most efficiently under mild acid stress conditions (pH 4.0–5.0) and are optimally induced during anaerobic conditions at pH > 5.0 (Kanjee and Houry, 2013). These conditions are consistent with the typical pH and anaerobic environment of the vagina during BV development (Ravel et al., 2011).

We hypothesize that BA production may mitigate the acidic barrier that favors vaginal lactobacilli. The BAs may also have a more direct effect on the growth of vaginal lactobacilli. A previous study revealed that the growth of several *Lactobacillus* species was stimulated by spermine and spermidine (Guirard and Snell, 1964), both of which we found to be significantly greater in the *L. crispatus*-dominated CST I and significantly lower in low-*Lactobacillus* CST IV. Additionally, elevated concentrations of putrescine as found in CST IV women in this study have previously been identified as inhibitors of growth of representative lactobacilli (Guirard and Snell, 1964).

BA production may also be important to other morbidities associated with BV, including increased risks of various STIs. Previous studies have shown host defenses to be less effective in the presence of BAs. Goytia and Schafer (Goytia and Shafer, 2010) revealed that the genital tract pathogen, *N. gonorrhoeae*, was more resistant to the impact of host mediated defenses in the presence of the BA, spermine. Similarly, the growth of *Legionella pneumophila* is enhanced in the presence of cadaverine, putrescine, spermine, and spermidine (Nasrallah et al., 2011), and expression of the virulence loci of *Salmonella typhimirium* is stimulated in the presence of putrescine and spermidine (Jelsbak et al., 2012). BAs have also been shown to initiate biofilm formation in a number of relevant disease pathogens, including *Vibrio cholera, Yersinia pestis, E. coli*, and *N. gonorrhoeae* (Patel et al., 2006; McGinnis et al., 2009; Parker et al., 2012; Sakamoto et al., 2012; Goytia et al., 2013; Karatan and Michael, 2013). Biofilms are a significant mechanism of disease and have been associated with BV (Swidsinski et al., 2013).

Various studies have previously described the presence of BAs in the vagina (Chen et al., 1979, 1982; Sanderson et al., 1983; Wolrath et al., 2002; Yeoman et al., 2013), wherein BAs have been associated with the characteristic vaginal odor of BV (Srinivasan et al., 2012; Yeoman et al., 2013). Consequently, they have been considered as a potential biomarker of BV (Chen et al., 1982; Wolrath et al., 2002; Sobel et al., 2012). Two recent studies have identified correlative relationships between particular vaginal taxa and either odor (Srinivasan et al., 2012; Yeoman et al., 2013) or directly to the relative concentration of certain BAs (Yeoman et al., 2013). Srinivasan et al. (2012) found associations between *Eggerthella* spp., *Leptotrichia amnionii, G. vaginalis, D. micraerophilus, Prevotella bivia, P. disiens, Porphyromonas asaccharolytica, P. micra*, and BVAB1 (BV-associated bacteria 1) each being correlated with clinically diagnosed vaginal odor. Several of these same genera (*Dialister* spp. with both putrescine and cadaverine; *Prevotella* spp., and *Porphyromonas* spp. with putrescine) were found to have Spearman's rank order correlations with specific BAs in the study of Yeoman et al. (2013). The same study also found strong correlations between both *Streptococcus* spp. and *Mycoplasma* spp. and cadaverine and between both *Mobiluncus* spp. and *Anaeroglobus* spp. with putrescine. Macklaim et al. (2013) in their recent investigation of the vaginal meta-transcriptome detected the expression of an arginine decarboxylase (putrescine) by *P. amnii*, and spermidine synthase genes by *Dialister* and *Megasphaera* genera. However, the only study providing physiological evidence for the production of a BA, TMA, revealed it to be produced by vaginal *Mobiluncus* species, and not by vaginal *Bacteroides* or *Gardnerella* species (Cruden and Galask, 1988). Our analyses support several of these correlative associations and all of the transcriptional observations of Macklaim et al. (2013). Discrepancies between correlative relationships and the genetic ability to biosynthesize BAs (i.e., correlations with *G. vaginalis, Streptococcus, Anaeroglobus*, and *Mobiluncus*) illustrate the limitations of correlative analyses and the difficulties in separating cause and effect. However, our findings are not consistent with the findings of Cruden and colleagues (Cruden and Galask, 1988). It is more difficult to reconcile this discrepancy, though it may be explainable by alternate and currently unrecognized pathways for the biosynthesis of TMA from either TMAO or choline or perhaps the trimethylamine N-oxide reductase and choline trimethylamine-lyase genes present in the *Mobiluncus* are sufficiently diverged to escape our bioinformatic approach. In either case, this is worthy of further investigation. Our results did support TMA production by a variety of other species, through the metabolism of TMAO, choline, and N,N,N-trimethylglycine, and from ergothione. We also observed weaker but full length matches in various *Veillonella* and *Janthinobacterium* spp., *Prevotella buccalis, P. disiens*, and *Acinetobacter baumanii* that may worthy of further interrogation.

Our additional findings demonstrate that several BA-producing taxa are enriched within the low-*Lactobacillus*, CST IV vaginal microbiome. In addition, taxa including *Prevotella, Megasphaera, Parvimonas*, and *Veillonella* spp. are found in maximum abundance when pH exceeds pH 4.6. Threshold analysis suggests a community change point occurs at pH 4.4 which displays a correlation between decreasing abundances of *Lactobacillus* spp. and increasing abundances of BV-associated bacteria with many of these taxa identified as BA-producers. This supports the hypothesis that BA production is an important factor for the mitigation of one of the most widely described barriers to vaginal pathogens, vaginal pH. Although *Lactobacillus* spp. are predicted to synthesize putrescine, we also show that cadaverine and tyramine are enriched when *Lactobacillus* spp. are depleted (CST IV), while spermine and spermidine are enriched within other CSTs.

Based on the data provided in this study and other observations in the literature, we hypothesize that the microbial production of BAs is more than just a biomarker for BV. We put forward a novel conceptual model of the role of BAs in the vagina and suggest they are an important metabolic feature for overcoming pH and facilitating the outgrowth of BV-associated bacteria. For women categorized as CST IV, the production of BAs by BV-associated vaginal taxa was more common than what we observed in other CSTs. This may represent a vulnerable CST for the vagina, whereby it is at a greater risk of colonization by microbe(s) capable of eliciting the additional signs or symptoms of BV. This may include subpopulations of *G. vaginalis*, who have been repeatedly linked to BV, are able to recapitulate the clue cell symptom in murine models (Gilbert et al., 2013), and are the major colonizing bacterial taxa of clue cells in humans (Cook et al., 1989). We also hypothesize that the agents that are capable of eliciting symptoms of BV would not be able to competitively colonize the more typical *Lactobacillus*-dominated vaginal ecosystem, consistent with the existing dogma. The current understanding of BV lacks a clear, mechanistic pathway for its development. The hypothesis proposed herein suggests the microbial synthesis of BAs has a major role in predisposing the vagina to BV development. In support of our hypothesis, we have shown through the analysis of available datasets, that genes encoding enzymes predicted to be capable of synthesizing BAs are present in a number of taxa. The bioinformatics approach used in this study provides a window into the potential of these bacteria to produce BAs, with limitations in our ability to accurately determine the activity of these genes. For this reason, this information warrants further investigations with culture-based determination of enzymes and BAs from bacteria relevant to the vaginal microbiome and associated with BV. One curiosity was the determination of putative genes capable of putrescine-production in many lactobacilli with an opposite correlation in our observation with metabolite data associated with CST groupings. Overall, we observed that the production of BAs is more common in the low-*Lactobacillus* CST IV, also termed “asymptomatic BV.” Taking this data together, we hypothesize that the production of BAs in the vagina increases the ability of BA-producing taxa to competitively colonize the vagina, putting the host at a greater risk of BV development. These findings may help to reshape our understanding of BV and shed a mechanistic light on particular microbes that may be important to vaginal dysbiosis, potentially at the earliest stage of BV.

### Conflict of interest statement

The authors declare that the research was conducted in the absence of any commercial or financial relationships that could be construed as a potential conflict of interest.

## Supplementary material

Figure S1 **Abundance trends of vaginal bacteria in relation to vaginal pH**. Graphs display the average relative abundance of Lactobacillus spp. and bacteria associated with bacterial vaginosis as pH increases. Abundance data was taken from Ravel et al. (2011).

Table S1 **Characterized homologs and HMMs utilized in this study**.

Table S2 **TITAN results for individual taxa response to pH**.
