# Supplementary figures and images for: Vaginal biogenic amines: biomarkers of bacterial vaginosis or precursors to vaginal dysbiosis?
Source: Front Physiol. 2015 Sep 29;6:253. doi: 10.3389/fphys.2015.00253 (PMC4586437; doi:10.3389/fphys.2015.00253)

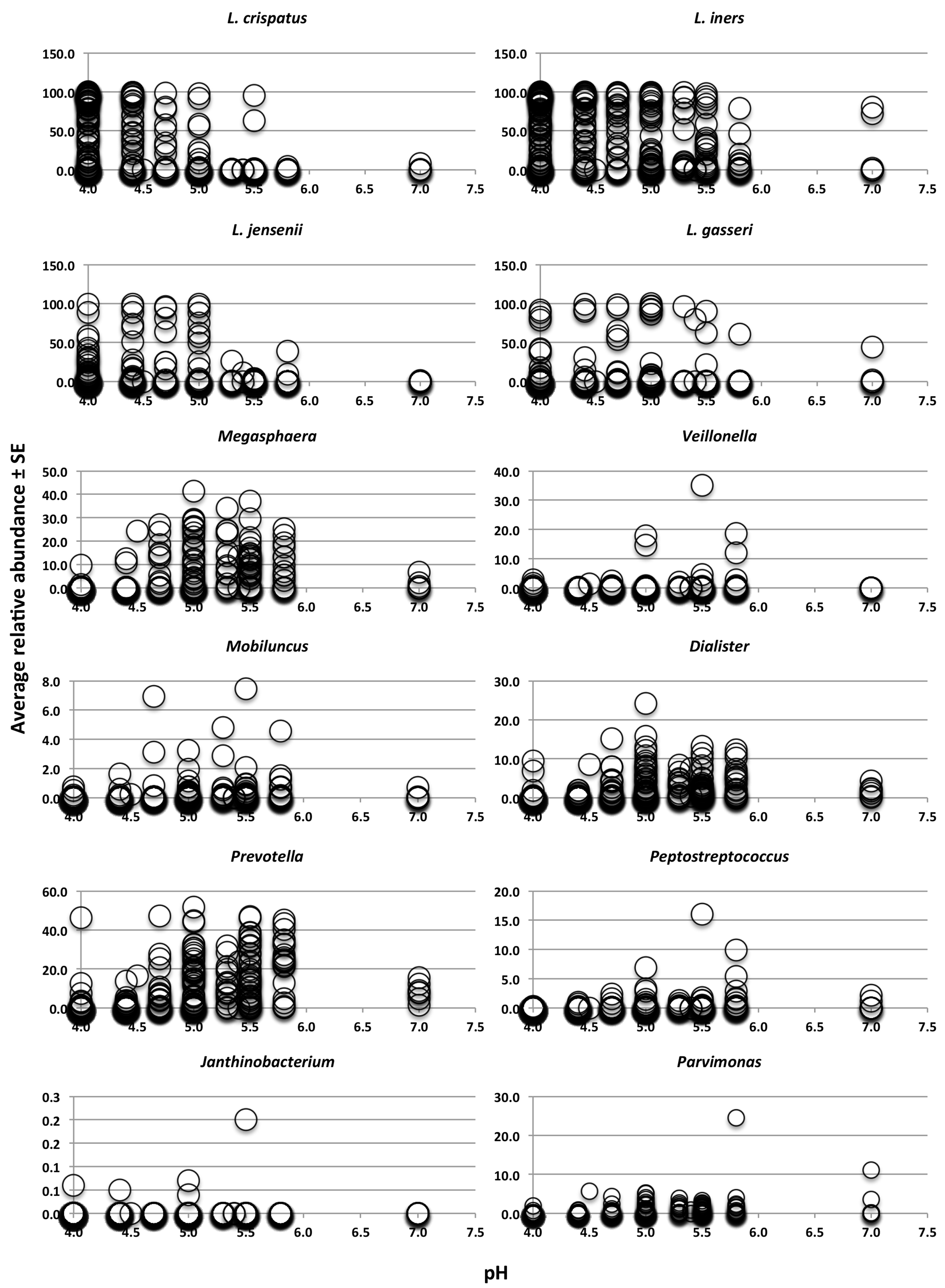

Supplement: Figure S1 — Abundance trends of vaginal bacteria in relation to vaginal pH. Graphs display the average relative abundance of Lactobacillus spp. and bacteria associated with bacterial vaginosis as pH increases. Abundance data was taken from Ravel et al. (2011). [file Image1.TIF]
