# Supplementary material for: Vaginal biogenic amines: biomarkers of bacterial vaginosis or precursors to vaginal dysbiosis?
Source: Front Physiol. 2015 Sep 29;6:253. doi: 10.3389/fphys.2015.00253 (PMC4586437; doi:10.3389/fphys.2015.00253)
Supplement: Table S1 — Characterized homologs and HMMs utilized in this study. [file Table1.DOCX]

Table S1. Characterized homologues and HMMs utilized in this study

| Biogenic amine-synthesizing protein | Characterized Homologue^1^ | Hidden Markov Models^2^ |
| --- | --- | --- |
| Ornithine decarboxylase (speF; E.C. 4.1.1.17) | *Lactobacillus* sp. 30a [65];  AFC60624 (*Lactobacillus brevis* IOEB 9906 ([83]);  CBY85125 (*Oenococcus oeni* [84]);  CAL36957 (*Morganella morganii* [85]);  *Pseudomonas aeruginosa* PAO1 [86] | TIGR04301 or TIGR04318^3^ |
| Arginine decarboxylase (speA; E.C. 4.1.1.19) | AAD07486 (speA; *Helicobacter pylori* 26695 [87]);  NP_225226 (aaxB; *Chlamydia pneumoniae* [88]^4^);  NP_253526 (*Pseudomonas aeruginosa* PAO1 [86]) | TIGR01273 |
| Agmatinase (E.C. 3.5.3.11) | WP_010884203 (*Pyrococcus horikoshii* [89]) | TIGR01230 |
| Agmatine deiminase (E.C. 3.5.3.12) | AguA (*Pseudomonas aeruginosa* PAO1 [86]; partial characterization);  (*Helicobacter pylori* [90]); | TIGR03880 |
| N-carbamoylputrescine amidohydrolase (E.C. 3.5.1.53) | NP_248984 (AguB; *Pseudomonas aeruginosa* PAO1 [86]; partial characterization) | TIGR03381 |
| Lysine decarboxylase (E.C. 4.1.1.18) | BAP47521 (*Burkholderia* sp. AIU 395 [91]);  AB011029 (*Selenomonas ruminantium subsp. Lactilytica* [92]^5^  BAA21656 (ldcC; *Escherichia coli* K12) [93]) | TIGR(01048) |
| Spermidine synthase (E.C. 2.5.1.6) | SPEE_HELPY (speE; *Helicobacter pylori* 26695 [94]) | TIGR00417 |
| Spermine synthase (E.C. 2.5.1.22) | AAC19368 (*Saccharomyces cerevisiae* 2602 [95]) |  |
| Tyrosine decarboxylase (E.C. 4.1.1.25) | AGW24519 (*Enterococcus faecalis* R612Z1 [96])  AFP73381 (*Lactobacillus brevis* GGMCC [97])  Q60358 (*Methanocaldococcus jannaschii* Q60358 [98]) | TIGR03811 |
| Trimethylamine N-oxide reductase (EC 1.7.2.3) | EDU64639 (*E. coli* K10 [99]^6^) | TIGR02164 |
| Choline Trimethylamine-lyase (4.3.99.4) | ACL49259 (*Desulfovibrio desulfuricans* ATCC 27774 [28]) | TIGR04394 |
| Betaine reductase (E.C. 1.21.4.4) | (*Eubacterium acidaminophilum* [100]) | Pfam09338 |
| Ergothionase | BAM63550 (*Burkholderia* sp. HME13 [29]) | N/A^7^ |

^1^GenBank accession number is listed, organism of origin and reference to characterization is also provided in brackets; ^2^HMMs are from the following resources – prefix TIGR = TIGERFAM; pfam = Pfam (http://pfam.xfam.org/); ^3^TIGR04318 is a *Lactobacillus*-specific HMM for ornithine decarboxylases. It did not hit any non-*Lactobacillus*, but all noted *Lactobacillus*. TIGR04301 did not match any *Lactobacillus* homologues. ^4^aaxB was reported as having an identical sequence to the heterologously characterized protein. ^5^Gene was extracted from larger region based on information provided in characterization paper and translated. ^6^Gene described in characterization study was not available in GenBank so a homologue in another *E. coli* strain was utilized. ^7^Ergothionase was only recently discovered and does not have an associated HMM or associated E.C. number. All biogenic amine synthesizing protein sequences are available in supplementary materials.
