# Supplementary material for: Vaginal biogenic amines: biomarkers of bacterial vaginosis or precursors to vaginal dysbiosis?
Source: Front Physiol. 2015 Sep 29;6:253. doi: 10.3389/fphys.2015.00253 (PMC4586437; doi:10.3389/fphys.2015.00253)
Supplement: Table S2 — TITAN results for individual taxa response to pH. [file Table2.DOCX]

Table S2. TITAN results for individual taxa response to pH

|  | **Threshold** | **Frequency** | **IndVal** | **P-value** | **z** | **5%** | **50%** | **95%** |
| --- | --- | --- | --- | --- | --- | --- | --- | --- |
| **z-** |  |  |  |  |  |  |  |  |
| Clostridium | 4 | 20 | 17.78 | 0.004 | 10.94 | 4 | 4 | 4.7 |
| Lactobacillus_2 | 4 | 48 | 17.55 | 0.004 | 7.96 | 4 | 4.4 | 4.7 |
| Lactobacillales_6 | 4.4 | 122 | 43.55 | 0.004 | 17.21 | 4 | 4.4 | 4.7 |
| L. vaginalis | 5 | 137 | 43.47 | 0.004 | 14.25 | 4.4 | 4.7 | 5 |
| **L. jensenii** | **4.4** | **190** | **41.37** | **0.004** | **9.1** | **4** | **4.4** | **5** |
| Lactobacillales_2 | 5 | 205 | 40.4 | 0.004 | 7.49 | 4.4 | 4.7 | 5.3 |
| **L. crispatus** | **4.7** | **254** | **62.52** | **0.004** | **16.14** | **4.4** | **4.45** | **5** |
| Lactobacillales_5 | 4.4 | 255 | 54.9 | 0.004 | 13.18 | 4.4 | 4.7 | 5 |
| **L. iners** | **4.7** | **329** | **50.81** | **0.004** | **5.24** | **4.4** | **5** | **5.8** |
| **z+** |  |  |  |  |  |  |  |  |
| **Prevotella** | **4.7** | **270** | **76.8** | **0.004** | **23.29** | **4.4** | **4.7** | **5** |
| **Dialister** | **4.7** | **206** | **70.47** | **0.004** | **25.2** | **4.4** | **4.7** | **5** |
| Peptoniphilus | 5 | 203 | 69.74 | 0.004 | 22.38 | 4.4 | 4.7 | 5 |
| Anaerococcus | 4.7 | 210 | 60.61 | 0.004 | 15.22 | 4.4 | 4.7 | 5 |
| Atopobium | 4.6 | 159 | 55.94 | 0.004 | 19.82 | 4.4 | 4.7 | 5 |
| Sneathia | 5 | 120 | 55.52 | 0.004 | 20.54 | 4.7 | 5 | 5 |
| Finegoldia | 5 | 197 | 54.78 | 0.004 | 12.68 | 4.4 | 4.7 | 5.3275 |
| **Megasphaera** | **4.7** | **139** | **51.46** | **0.004** | **20.27** | **4.4** | **4.7** | **5** |
| Prevotellaceae_2 | 5 | 92 | 50.47 | 0.004 | 19.21 | 4.7 | 5 | 5.3 |
| Bacteroides | 5.8 | 70 | 47.07 | 0.004 | 10.05 | 5 | 5.5 | 5.8 |
| **Parvimonas** | **5** | **106** | **46.23** | **0.004** | **20.83** | **4.4** | **4.7** | **5.3** |
| Gardnerella | 4.7 | 131 | 46.21 | 0.004 | 15.88 | 4.4 | 4.7 | 5 |
| Porphyromonas | 5 | 93 | 44.94 | 0.004 | 16.98 | 4.7 | 5 | 5.3 |
| Eggerthella | 4.7 | 99 | 43.75 | 0.004 | 21.85 | 4.7 | 4.775 | 5 |
| Gemella | 5 | 92 | 43.61 | 0.004 | 19.2 | 4.4225 | 5 | 5.3 |
| Moryella | 5.5 | 45 | 42.07 | 0.004 | 14.14 | 5 | 5.3 | 5.8 |
| Corynebacterium | 4.7 | 173 | 41.92 | 0.004 | 8.73 | 4.4 | 5 | 5.5 |
| Streptococcus | 4.4 | 146 | 40.9 | 0.004 | 10.44 | 4 | 4.4 | 4.7 |
| Ruminococcaceae_3 | 4.7 | 92 | 40.83 | 0.004 | 20.53 | 4.45 | 5 | 5.3 |
| Aerococcus | 4.7 | 114 | 40.31 | 0.004 | 14.14 | 4.4 | 4.7 | 5 |
| Fusobacterium | 5.5 | 42 | 39.39 | 0.004 | 14.49 | 4.7 | 5.5 | 5.8 |
| Anaeroglobus | 4.7 | 79 | 38.21 | 0.004 | 18.42 | 4.7 | 5 | 5 |
| Clostridiales_17 | 5.3 | 81 | 37.81 | 0.004 | 12.03 | 4.7 | 5 | 5.5 |
| **Mobiluncus** | **4.7** | **90** | **36.39** | **0.004** | **15.53** | **4.4** | **4.7** | **5.3** |
| **Peptostreptococcus** | **5** | **89** | **36.15** | **0.004** | **12.84** | **4.4** | **4.7** | **5.5** |
| Enterobacter | 5.8 | 21 | 32.31 | 0.004 | 12.1 | 4.7 | 5.8 | 7 |
| Ruminococcaceae_4 | 5 | 69 | 31.73 | 0.004 | 13.03 | 4.7 | 5 | 5.3 |
| Actinomyces | 5 | 64 | 30.57 | 0.004 | 13.4 | 4.5175 | 5 | 5.5 |
| Ruminococcaceae_Incertae_Sedis | 5 | 61 | 30.44 | 0.004 | 13.61 | 4.4 | 5 | 5 |
| Propionibacterium | 5.8 | 38 | 30.38 | 0.004 | 6.96 | 4.4 | 5.325 | 5.8 |
| Campylobacter | 5 | 65 | 27.92 | 0.004 | 13.46 | 4.7 | 5 | 5.5 |
| Prevotellaceae_1 | 5 | 58 | 27.76 | 0.004 | 14.64 | 4.7 | 5 | 5.3 |
| Mycoplasmataceae_1 | 5 | 44 | 26.7 | 0.004 | 14.14 | 4.7 | 5.3 | 5.8 |
| Peptococcus | 5.5 | 44 | 26.49 | 0.004 | 11.2 | 4.7 | 5.3 | 5.8 |
| Arcanobacterium | 5 | 47 | 24.06 | 0.004 | 11.89 | 4.7 | 5 | 5.3 |
| Lachnospiraceae_7 | 4.7 | 58 | 23.27 | 0.004 | 10.87 | 4.4 | 4.7 | 5.5 |
| Segniliparus | 4.7 | 66 | 23.02 | 0.004 | 9.18 | 4.4 | 5 | 5.5 |
| Lactobacillales_7 | 5 | 74 | 22.85 | 0.004 | 8.69 | 4.4 | 4.7 | 5 |
| Coriobacteriaceae_2 | 4.7 | 48 | 22.11 | 0.004 | 12.89 | 4.4 | 4.7 | 5 |
| Lachnospiraceae_8 | 4.7 | 53 | 21.91 | 0.004 | 10.7 | 4.4 | 4.7 | 5 |
| Bacteroidetes_8 | 4.7 | 41 | 21.59 | 0.004 | 12.41 | 4.4 | 5 | 5.4775 |
| Bulleidia | 5 | 35 | 21.45 | 0.004 | 12.27 | 4.4 | 5 | 5.5 |
| Anaerovorax | 5.5 | 27 | 20.53 | 0.004 | 8.45 | 4.535 | 5.5 | 6.73 |
| **Veillonella** | **4.7** | **58** | **20.18** | **0.004** | **7.89** | **4.4** | **5** | **5.5** |
| Gallicola | 5.5 | 22 | 18.79 | 0.004 | 8.98 | 5 | 5.5 | 6.13 |
| Bacteroidales_1 | 5.3 | 31 | 18.33 | 0.004 | 10.39 | 4.7 | 5 | 5.5 |
| Varibaculum | 4.4 | 44 | 16.17 | 0.004 | 7.9 | 4.4 | 4.7 | 5.8 |
| Clostridiales_6 | 5.5 | 17 | 15.94 | 0.004 | 10.65 | 5 | 5.3 | 5.8 |
| Proteobacteria_12 | 5 | 38 | 15.75 | 0.004 | 9.16 | 4.4 | 4.7 | 5.3 |
| Lachnospiraceae_4 | 4.7 | 31 | 15.42 | 0.004 | 9.32 | 4.7 | 5 | 5.165 |
| Clostridiales_8 | 5.5 | 7 | 13.8 | 0.004 | 11.44 | 5.3 | 5.5 | 5.8 |
| Fastidiosipila | 5 | 28 | 13.36 | 0.004 | 9.44 | 4.7 | 5.5 | 7 |
| Clostridiales_15 | 4.7 | 28 | 13.24 | 0.004 | 8.95 | 4.7 | 5.3 | 5.5 |
| Sutterella | 4.7 | 33 | 11.73 | 0.004 | 5.86 | 4.4 | 4.7 | 5.8 |
| Lachnospiraceae_9 | 4.7 | 23 | 11.72 | 0.004 | 9.22 | 4.7 | 5 | 5 |
| Clostridiales_4 | 5 | 19 | 11.71 | 0.004 | 8.38 | 4.7 | 5 | 5.5 |
| Coriobacteriaceae_3 | 5 | 20 | 11.71 | 0.004 | 9.52 | 4.7 | 5.3 | 5.5 |
| Dethiosulfovibrio | 5.5 | 16 | 11.37 | 0.004 | 4.44 | 4.4 | 5.3 | 7 |
| Bacteroidetes_12 | 4.7 | 17 | 11.11 | 0.004 | 8.67 | 4.7 | 5 | 5.8 |
| Lachnospiraceae_2 | 5 | 21 | 10.98 | 0.004 | 7.59 | 4.7 | 5 | 5.5 |
| Proteobacteria_1 | 4.6 | 26 | 10.69 | 0.004 | 6.57 | 4.4 | 4.7 | 5.3 |
| Bacteroidetes_1 | 4.7 | 17 | 9.2 | 0.004 | 8.71 | 4.7 | 5 | 5.5 |
| Proteobacteria_2 | 4.4 | 22 | 9.17 | 0.004 | 5.77 | 4.4 | 4.7 | 5.3 |
| Lachnospiraceae_1 | 4.7 | 14 | 9.09 | 0.004 | 8.03 | 4.45 | 5 | 5.3 |
| Actinobaculum | 5 | 14 | 7.86 | 0.004 | 5.78 | 4.7 | 5 | 7 |
| TM7_genera_incertae_sedis | 4.4 | 15 | 6.2 | 0.008 | 4.64 | 4 | 4.4 | 7 |

Threshold indicator taxa analysis (TITAN) observed thresholds and bootstrap confidence intervals value of x resulting in the largest indicator value (IndVal) z scores for each taxon (see Figure 5). Based on 500 simulation iterations in which the 90% bootstrap confidence interval included the true threshold. Freq. is the number of non-zero observations. Bolded taxa represent those identified as possessing genes for biogenic amine-production.
